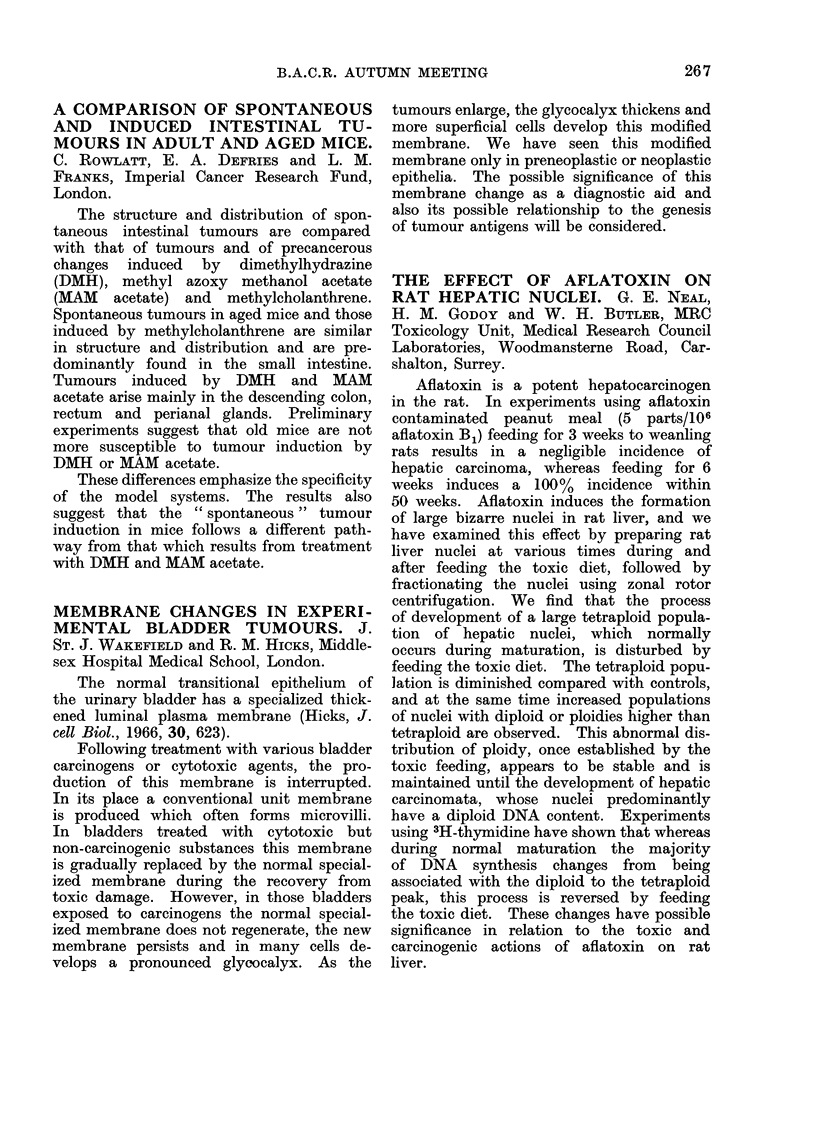# Proceedings: A comparison of spontaneous and induced intestinal tumours in adult and aged mice.

**DOI:** 10.1038/bjc.1975.59

**Published:** 1975-02

**Authors:** C. Rowlatt, E. A. Defries, L. M. Franks


**A COMPARISON OF SPONTANEOUS AND INDUCED INTESTINAL TUMOURS IN ADULT AND AGED MICE.** C. ROWLATT, E. A. DEFRIES and L. M. FRANKS, Imperial Cancer Research Fund, London.

The structure and distribution of spontaneous intestinal tumours are compared with that of tumours and of precancerous changes induced by dimethylhydrazine (DMH), methyl azoxy methanol acetate (MAM acetate) and methylcholanthrene. Spontaneous tumours in aged mice and those induced by methylcholanthrene are similar in structure and distribution and are predominantly found in the small intestine. Tumours induced by DMH and MAM acetate arise mainly in the descending colon, rectum and perianal glands. Preliminary experiments suggest that old mice are not more susceptible to tumour induction by DMH or MAM acetate.

These differences emphasize the specificity of the model systems. The results also suggest that the " spontaneous " tumour induction in mice follows a different pathway from that which results from treatment with DMH and MAM acetate.